# Bayesian Optimization of a Suzuki Coupling for the Industrial Synthesis of a Sartan Drug Intermediate

**DOI:** 10.1021/acsomega.6c04219

**Published:** 2026-08-03

**Authors:** Maite Molins, Javier Fernandez-Garcia, Daniel Vázquez, Xavier Berzosa

**Affiliations:** † CRISOL Group, Dept. of Chemical Engineering and Materials Science, IQS-School of Engineering, 667358 Universitat Ramon Llull, Via Augusta 390, 08017 Barcelona, Spain; ‡ GESPA Group, Dept. of Chemical Engineering and Materials Science, IQS-School of Engineering, Universitat Ramon Llull, Via Augusta 390, 08017 Barcelona, Spain

## Abstract

Sartans are an important family of drugs used to treat hypertension. A common characteristic of most of them is a biphenyl moiety, commonly obtained through a Suzuki-Miyaura cross-coupling reaction, of which thousands of tons are produced every year. Bayesian Optimization (BO), a machine-learning technique to find the optimum of complex unknown functions with a minimum number of test points, has been applied to optimize the reaction conditions in continuous flow with a focus on lowering the costs and increasing both the productivity and the sustainability of the process, as evaluated through its Process Mass Intensity (PMI). Although this is a common chemical transformation, the fact that it happens in a biphasic medium with organometallic catalysis makes its optimization more challenging. Six variables were identified as important for the reaction system: residence time, temperature, equivalents of the nonlimiting reactant, mol % of catalyst, mol % of ligand, and solvent volumes. With less than 40 experiments, the BO algorithm achieved solutions that increased the productivity up to 4 times compared to the initial results of the DOE experiments and reduced the cost by around 30%. A Pareto front with different optimal solutions was provided at the end of the optimization, and the point with the lowest cost and the lowest PMI was scaled up to increase productivity while keeping the unitary cost unchanged.

## Graphical abstract

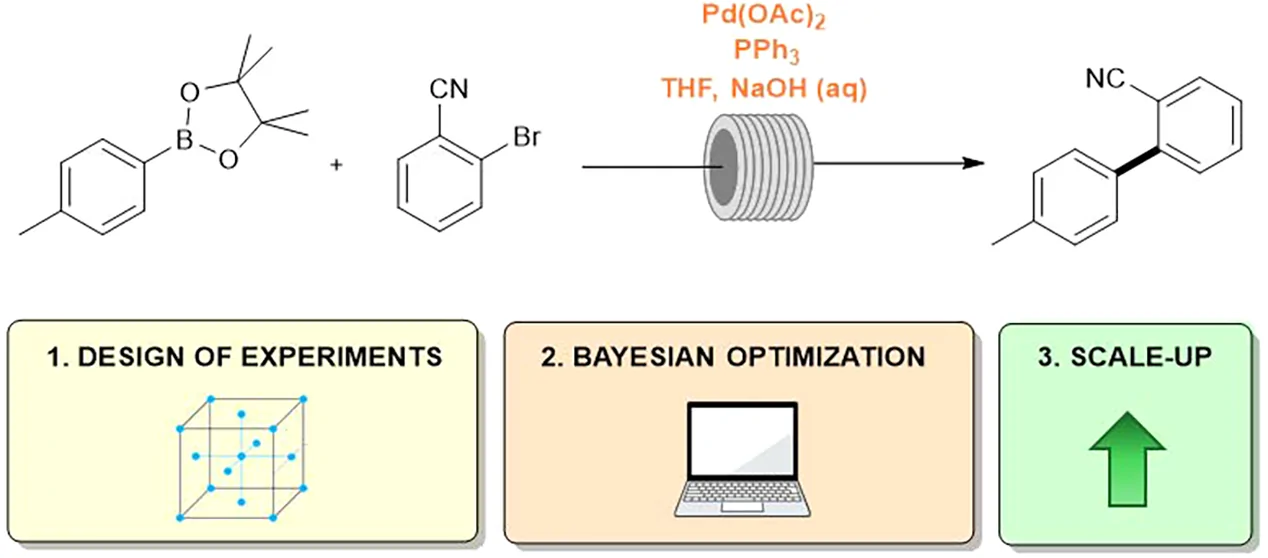

## 1 Introduction

Sartans are an important family of drugs used to treat hypertension, with a total global consumption of raw materials of 34,282 tons from 2016 to 2022.^1,2^ Sartan molecules typically share a scaffold composed of a diphenyl group with a tetrazole ring or an acid group in *ortho* position to the phenyl–phenyl bond (Figure 1).^3^ This biphenyl molecule, typically 2-cyano-4′-methylbiphenyl, is usually synthesized through a palladium-catalyzed Suzuki–Miyaura cross-coupling reaction, as reported in several articles, reviews and patents.^4–12^

**Figure 1:**
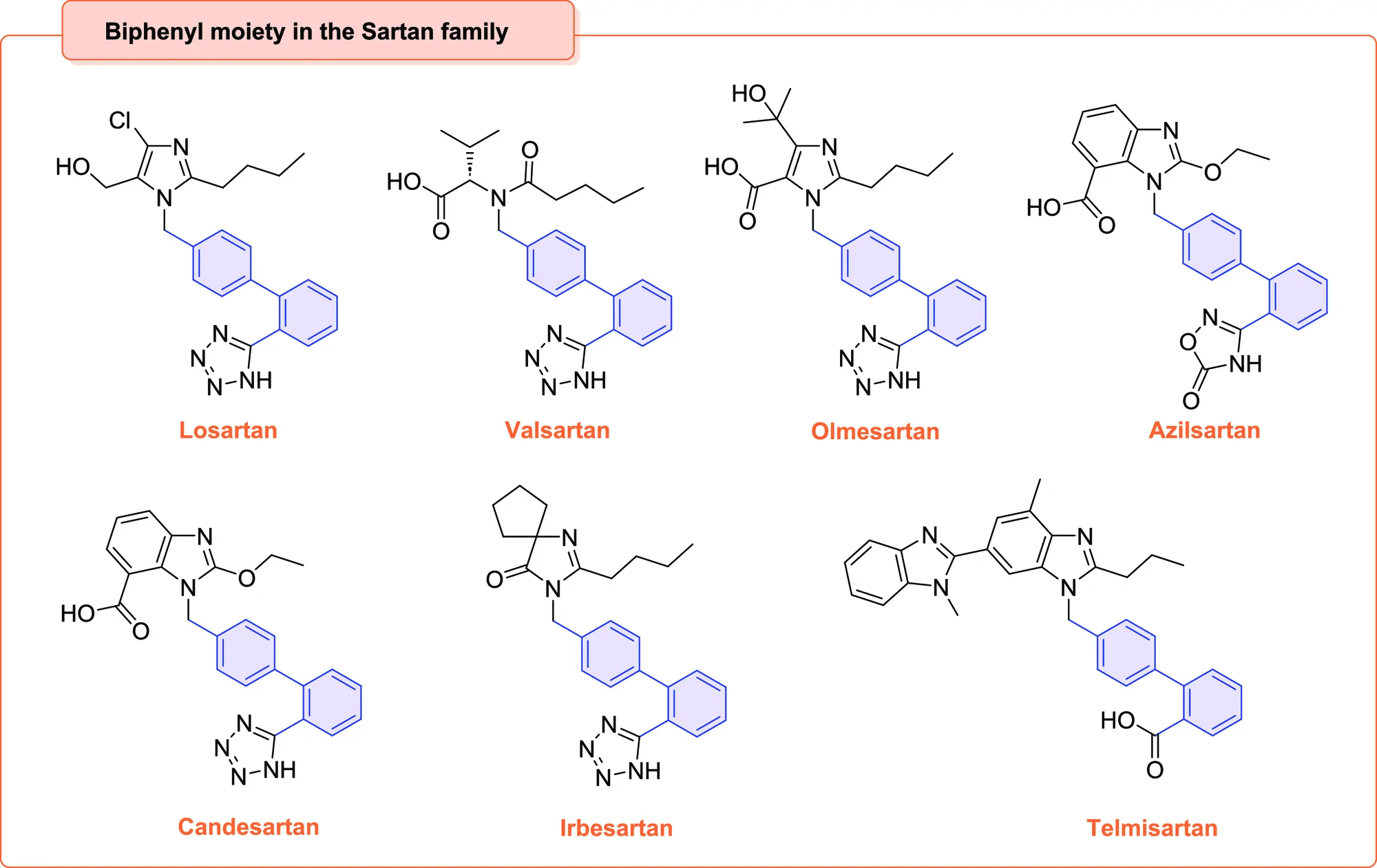
Molecules of the sartan family displaying the biphenyl ring, common in all of them.

The Suzuki–Miyaura cross-coupling reaction allows for the creation of a carbon–carbon bond between an alkenylborane (a boronic acid or a boronic ester) and an aryl halide under mild conditions, in the presence of a Pd catalyst (ligated or not) and a base.^13,14^ A thorough examination of Suzuki coupling reactions was conducted, focusing on those methodologies that had been reported to work on the kilogram scale. The most common ligand for Suzuki reactions in general is Tetrakis­(triphenylphosphine)­palladium, representing around 46% of the reported examples (SciFinder). However, when only kg-scale examples are considered, palladium diacetate is the most common due to higher reliability,^15^ frequently in the presence of PPh_3_ as a ligand.^16–21^

Several methods exist for the optimization of chemical reactions, from the simpler One-factor-at-a-time (OFAT) to the more complex Machine Learning and AI Algorithms, passing through Model Driven approaches such as the classical Design of Experiments strategies (DoE) or kinetic modeling.^22^ Although the OFAT approach is simple to understand, it is highly resource-consuming and ignores the interactions between variables. On the other hand, DoE approaches include interactions between variables but are time-consuming to perform when a large number of variables or levels need to be considered (as the number of experiments increases exponentially with the number of factors to study). Therefore, they are usually kept for the latest stages of process optimization.

Focusing on machine learning algorithms that minimize the number of experiments to perform while still exploring and exploiting the solution space, Bayesian optimization (BO) is especially adept at finding optima in expensive-to-evaluate functions.^23^ BO is a sequential approach that consists of building probabilistic models, specifically Gaussian Processes (GP) based on multivariate normal distributions, that are then optimized. The algorithm optimizes a composite function, denominated acquisition function, that encompasses both the desired predicted value based on the objective function and the uncertainty of the prediction built from the GP models. Moreover, BO can handle uncertainty intrinsically, which may be related to possible measurement noise of the used equipment during sampling.

In recent years, BO has been employed for the optimization of chemical reactions by several research groups, achieving significant efficiency gains with this method compared to other numerical or heuristic approaches.^24^ Examples of single-task and multitask BO to accelerate the optimization of chemical transformations have been reported,^25–28^ as well as the combination of BO with the Nelder–Mead simplex method,^29^ neural networks^30^ or fluid dynamics simulations.^31^ BO has also been used for kinetic parameter estimation.^32^ Moreover, BO algorithms are made available in “ready to use” open-source packages such as BoTorch and Ax (Python),^24,33–36^ where one can manage optimization problems easily, including laboratory experiments and constraints.

The combination of human input and expertise with optimization workflows is known as Human-in-the-loop optimization, and is especially effective when experimental testing is required.^37–39^ For example, BO algorithms do not have a clear stopping point, since the optimized acquisition functions include the uncertainty of the search space in them, and thus, there are no true optimums since this uncertainty changes after each iteration, as well as where the optimum of the surrogate model may be. Therefore, human input is necessary to establish the stopping criteria.

In this work, we use Human-in-the-loop BO as an efficient strategy to identify optimal operating conditions for the production of the key industrial intermediate 2-cyano-4′-methylbiphenyl (**3**) from the coupling of 4,4,5,5-Tetramethyl-2-*p*-tolyl-1,3,2-dioxaborolane (**1**) and *o*-bromobenzonitrile (**2**) under continuous flow (Scheme 1). Starting from a simple initial design space, BO provides a data-efficient and systematic exploration of the reaction conditions, significantly reducing the experimental effort typically required for process optimization. From an engineering perspective, the BO strategy simultaneously targets the minimization of costs and the maximization of productivity, while integrating sustainability-oriented metrics such as Process Mass Intensity (PMI)^40,41^ to assess the sustainability of the experimental conditions found. By incorporating these factors into the optimization workflow, the current approach provides a systematic pathway to identify manufacturing conditions that balance performance, cost, and sustainability, highlighting the significant potential of BO as a powerful tool for chemical process development.

**Scheme 1:**
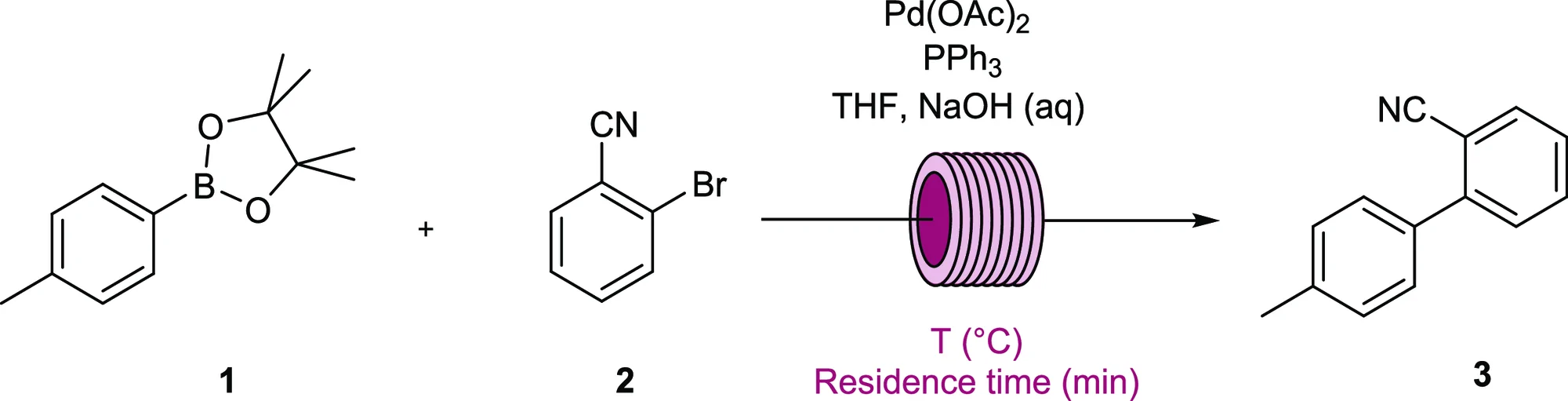
Reaction Scheme for the Suzuki Coupling to Form 2-Cyano-4′-methylbiphenyl

## 2 Experimental Section/Methods

### 2.1 Chemicals and Analysis

All reagents and solvents were purchased from standard suppliers (BLDpharm, Fluorochem, TCI, Fisher) and used without further purification. Crude analysis was carried out through NMR spectra recorded on a Jeol 400 MHz or a Jeol 600 MHz instrument (400 or 600 MHz for ^1^H). Chemical shifts are reported as δ-values in parts per million (ppm) and are referred to the partially deuterated isotopic solvent (chloroform: δ­[^1^H] = 7.26 ppm).

To estimate the cost of producing every gram of the target substance, the cost of the reagents and solvents has been checked at the Web site Chemicalbook (www.chemicalbook.com) and Imarc Group (www.imarcgroup.com) taking as a reference the price per kilogram for the largest container available (more details are provided in Table S1 in the Supporting Information).

### 2.2 Synthesis in Flow

#### 2.2.1 Optimization Experiments

Flow reactions were performed using a KD Scientific Syringe pump, where the reagents, the catalyst and the ligand in THF (organic phase) were loaded in a 10 mL syringe, and the inorganic phase (containing the aqueous NaOH) in another one. Both streams were mixed in a T-junction and circulated, for the appropriate residence time, through a 1 mL PTFE reactor (1/32″ ID and 1/16″ OD, Darwin Microfluidics) submerged in a thermal bath. A 10 psi back-pressure regulator was added at the outlet of the reactor to make sure THF remained liquid for the range of operation temperatures. After three residence times (to ensure stationary state), the samples were collected over concentrated HCl (37% w/w) to quench the reaction, for one residence time. 0.05 mmol of the internal standard 1,3,5-trimethoxybenzene were added to the reaction crude for quantification (Figure 2).

**Figure 2:**
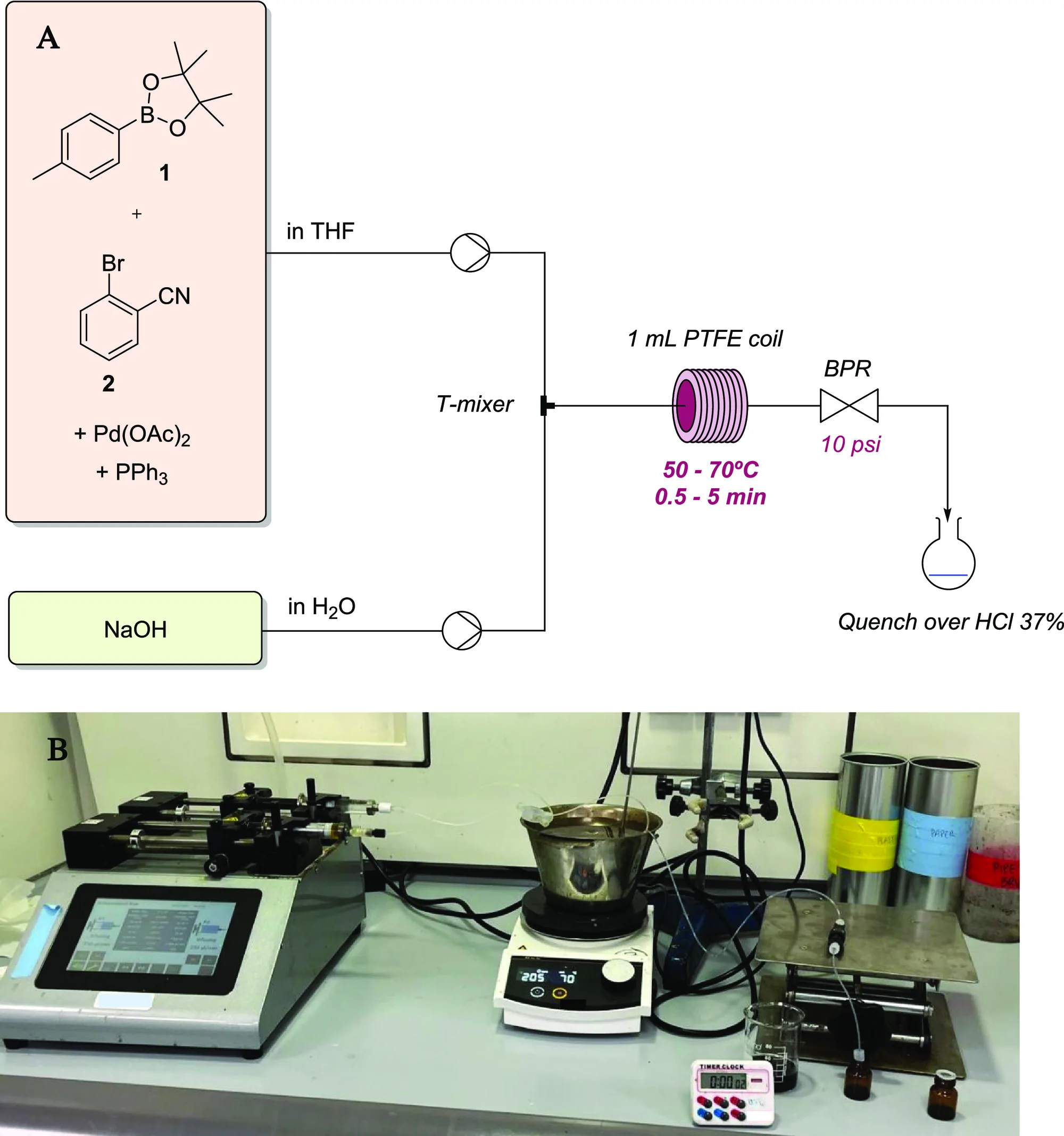
(A) Scheme of the flow setup employed in the reactions. (B) Photo of the actual setup used in the laboratory.

#### 2.2.2 Scale-Up

Flow scale-up was performed with a 5 mL PTFE reactor (1/16″ ID and 1/8″ OD, Darwin Microfluidics) coupled to two continuous syringe pumps (Syrris Asia and Infusetek Constinfuse). One of the syringe pumps injected the organic phase and the other one the aqueous NaOH. Both streams were mixed through a T-junction and passed through the reactor at the design temperature for the corresponding amount of time. Samples were collected in Erlenmeyer flasks over concentrated HCl (37%) to quench the reaction.

### 2.3 Bayesian Optimization

BO is a sequential procedure that allows for the optimization of an expensive-to-evaluate function by building a probabilistic surrogate model of the process based on experimental data. The goal is to determine an optimal or a near-optimal value after a small number of evaluations assuming that, in most cases, these evaluations will be affected by noise. The BO algorithms will be responsible for adaptively selecting the test points, balancing regions of good observed performance and regions of high uncertainty.

As the BO algorithms need to select a small number of test points to optimize the objective function, they must be able to predict what the function might look like at points that have not yet been evaluated, as well as quantifying the uncertainty of its predictions. This is referred to as a surrogate model and is in this case a Gaussian Process, for which the uncertainty is taken from a multivariate normal distribution.

Bayesian Optimization was implemented through the Python package BoTorch, where the GP is specified by a mean function μ­(*x*) and a covariance kernel *k*(*x*,*x* ^′^), from which a mean vector (μ­(*x*_0_),...,μ­(*x*_*k*_)) and covariance matrix Σ with Σ_*ij*_ = *k*(*x*_*i*_,*x*_*j*_) can be computed for any set of points (*x*_1_,...*x*_*k*_). Using a GP surrogate model for *f* means that we assume (*f*(*x*_1_),...,*f*(*x*_*k*_)) is multivariate normal with a mean vector and covariance matrix determined by μ­(*x*) and *k*(*x*,*x* ^′^). More information regarding the kernel and the hyperparameters can be found in the article by Hvarfner et al. (2024).^42^

The reported work is based on human-in-the-loop BO which worked as shown in Figure 3. An initial DoE, containing 16 distinct experimental conditions (X*) and their associated results (Y*), was performed in the laboratory to provide initial training data for the BO model. Once trained on this data, the BO model was combined with three acquisition functions (LEI: Log Expected Improvement, UCB: Upper Confidence Bound and PI: Probability of Improvement) each of which proposed a new, theoretical outcome (Y_opt,pred_*) for a given set of experimental conditions (X_opt_*). These predictions were validated in the laboratory, creating new data to train the model with. An important comment is that, if two acquisition functions suggested the same point, or points so similar that they could not be experimentally tested separately, it was only tested once. Alternatively, the batch mode of one of these acquisition functions could have been used, although the authors avoided that as no previous reference of which acquisition function would work best in the context of this work was present.

**Figure 3:**
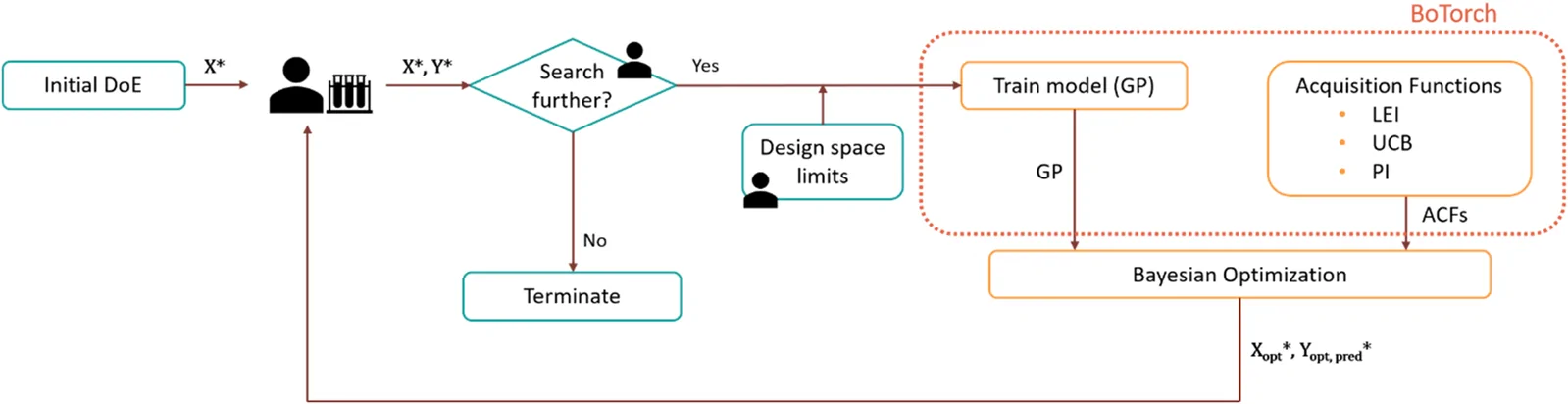
Human-in-the-loop Bayesian Optimization algorithm (LEI: Log Expected Improvement. UCB: Upper Confidence Bound. PI: Probability of Improvement. GP: Gaussian Processes. X: Decision variables. Y: Experimental Response).

The human interacted with the loop in two scenarios. First of all, if a parameter was kept in its higher or lower limit by the BO algorithm for more two rounds (and the optimal solution could fall outside of the initial search interval), the range was increased to allow the algorithm to test experimental conditions outside of the initial range, in case the optimal solution had not been included at the beginning. Second, the BO algorithm does not have a clear stopping point. Thus, in this work, the stopping criteria were established based on the difference between the new experimental conditions suggested by the model and the knowledge of the authors on whether conditions can still be improved by overexploiting certain regions.

#### 2.3.1 Variables of Interest

Three variables of interest are calculated for each set of conditions: unitary cost, productivity and PMI.

Unitary cost $\left(\mathrm{cost}\left[\frac{\$}{g}\right]\right)$ was calculated as the sum of the price of all starting materials and solvents (calculated by multiplying the mass consumed per its unitary cost), divided by the grams of product obtained (eq 1). The borylated starting material, *o-*bromobenzonitrile, palladium acetate, triphenylphosphine, THF, NaOH and water were included in the calculation. The cost of heating was initially considered, although it was removed early in the experimentation stage as it was negligible.

1 $$\mathrm{cost}=\frac{\sum_{i\in I}{m_{i}\cdot \mathrm{cost}}_{i}}{m_{\mathrm{product}}}$$

Where *i*∈*I* is the set of materials, *Cost*_*i*_ is the unitary price of each component $\left[\frac{\$}{g}\right]$ (see Table S1 in the Supporting Information), *m*_*i*_ [*g*] is the mass of each material and *m*_product_ [g] is the mass of product obtained from the reaction.

The mass of product obtained from the reaction (*m*_product_ [g]) was calculated from the mass of the limiting reagent pumped *m*_lim_ [g], the molar mass of both the limiting reagent and the product $\left(M_{\lim},M_{\mathrm{product}}\left[\frac{g}{\mathrm{mol}}\right]\right)$ , and the molar yield (η) of the transformation as illustrated in eq 2.

2 $$m_{\mathrm{product}}=m_{\lim}\cdot \frac{M_{\mathrm{product}}}{M_{\lim}}\cdot \eta$$

Productivity $\left(P\left[\frac{g}{h}\right]\right)$ was calculated considering the flow rate of organic solution pumped $\left(Q_{\mathrm{org}}\left[\frac{\mathrm{mL}}{h}\right]\right)$ , the concentration of the limiting reagent in the organic solution at the reactor inlet $\left(C_{\lim}\left[\frac{g}{\mathrm{mL}}\right]\right)$ , the molar mass of both the limiting reagent and the product $\left(M_{\lim},M_{\mathrm{product}}\left[\frac{g}{\mathrm{mol}}\right]\right)$ , and the reaction molar yield (η), as shown in eq 3.

3 $$P=Q_{\mathrm{org}}\cdot C_{\lim}\cdot \frac{M_{\mathrm{product}}}{M_{\lim}}\cdot \eta$$

PMI was calculated considering the mass of all materials (where *i*∈*I* is the set of materials) divided by the mass of product obtained, as displayed in eq 4.

4 $$\mathrm{PMI}=\frac{\sum_{i\in I}m_{i}}{m_{\mathrm{product}}}$$

#### 2.3.2 Objective Functions

Two objective functions (*OF*_1_,*OF*_2_) were defined to look for the best solutions in terms of cost and productivity (the best solutions would be the ones that minimize the value of the objective functions). Regarding the specific objective functions, we defined *OF*_1_ as shown in eq 5. *OF*_1_ represents the unitary cost $\left[\frac{\$}{g}\right]$ with a penalization on productivities $P\left[\frac{g}{h}\right]$ lower than 3 g/h by introducing a large value in the objective function (10 $/g). Given that the majority of the starting points in the DoE did not satisfy the constraint, the penalty was not introduced until Round 23, once enough points that satisfied the constraint were present. Further information on how the optimization was performed and developed can be found in Sections 2 and 3 in the Supporting Information.

5 $$OF_{1}\left(x\right)=\{\begin{array}{ll} \mathrm{cost} & P\geq 3g/h\\ 10\$/g & P<3g/h \end{array}$$

For *OF*_2_, we considered a weighted average approach including both unitary cost and productivity with the same weights. The equation was written in such a way that an increase in productivity and a decrease in cost would lead to the minimization of *OF*_2_, as shown in eq 6.

6 $$OF_{2}\left(x\right)=\mathrm{cost}-P$$

#### 2.3.3 Acquisition Functions and Optimization Model

Acquisition functions are heuristics used to evaluate how useful one or more design points are for optimizing the underlying black box function. In this article, analytic acquisition functions have been used to provide, in each case, a single candidate point. The acquisition functions used were: Expected Improvement (in its log form, LEI),^43^ Upper Confidence Bound (UCB) and Probability of Improvement (PI). More information about the functions and the BoTorch package can be found at https://botorch.readthedocs.io.

The objective functions *OF*_1_, *OF*_2_ were fed to specific acquisition functions preprogrammed in BoTorch to perform the BO. For each objective function, and through the application of the three different acquisition functions, three different new sampling points were proposed by solving the following optimization problem (eq 7)­

7 $$\min_{x}\left\{\mathrm{LEI}\left(x,f\right),\mathrm{UCB}\left(x,f\right),\mathrm{PI}\left(x,f\right)\right\}x\in R^{n},,f\in \left\{OF_{1},OF_{2}\right\}$$

Where *x* refer to the variables studied, i.e., residence time, temperature, equivalents of the nonlimiting reagent, mol % of catalyst, mol % of ligand and solvent volumes (mL of solvent per gram of limiting reagent). At each iteration, these solutions are tested in the laboratory and added to the surrogate model, which is then retrained.

## 3 Results and Discussion

The optimization of the Suzuki–Miyaura cross-coupling started with a two-level fractional factorial design of Experiments (DoE) to produce the initial data to feed to the BO algorithm using the software Minitab 17. As a compromise between the experimental effort and design resolution, a 2^6–2^ fractional factorial design was selected, reducing the number of experiments from 64 to 16 while keeping the main factors and first-order interactions unconfounded. The factors considered (Table 1) included residence time, temperature, equivalents of 2-bromobenzonitrile, mol % of catalyst (Pd­(OAc)_2_), mol % of ligand (PPh_3_) and solvent volumes, and the output was analyzed for three different variables: Cost, Productivity and PMI.

**Table 1:** Factors Considered in the DoE, Displaying the Two Levels

factor	–	+
Residence time	1 min	5 min
Temperature	50 °C	70 °C
Equiv. of 2-bromobenzonitrile	1.05	1.20
mol % of Pd(OAc)_2_	0.01	0.05
mol % of PPh_3_	0.01	0.05
Solvent volumes	10	20

The 16 points of the DoE plus the center point were fed to the BO algorithm. The residence time and the solvent volumes were identified in the initial DoE as high-impact reaction parameters, and as such had their ranges expanded to include lower amounts of solvent as well as lower residence times. The same was done for the catalyst, as it was the most expensive component in the reaction. The ranges initially fed to the BO algorithm are displayed in Table 2. All the initial results of the DoE are provided in Tables S2 and S3 (Supporting Information).

**Table 2:** Initial Design Space for the BO Algorithm

factor	min	max
Residence time	0.5 min	5 min
Temperature	50 °C	70 °C
Equiv. of 2-bromobenzonitrile	1.05	1.20
mol % of Pd(OAc)_2_	0.005	0.05
mol % of PPh_3_	0.01	0.05
Solvent volumes	5	20

The BO algorithm was executed several times to provide new experiments that would minimize the objective function. The two objective functions, *OF*_1_ and *OF*_2_, were used and run independently. For each new iteration, the three acquisition functions described previously were run and therefore three new points were tested.

In Figure 4 the evolution of Cost and Productivity as BO progresses is shown, both when optimizing *OF*_1_ and *OF*_2_ (specific values are included in Tables S2 and S3 in the Supporting Information). In general, most points suggested by the Bayesian Optimization algorithm outperformed the ones found in the DoE, showing higher productivity (of up to 9.6 g/h in a 1 mL reactor, which is a 400% increase compared to the best value in the initial DoE) and lower costs (of even under 1 $/g, a 27% lower than the best value in the initial DoE). Although the absolute prices are only an estimation, as actual prices from industrial-scale manufacturers would be lower, the comparison between initial and final prices shows how BO optimization can have a significant impact in the reduction of costs in the process.

**Figure 4:**
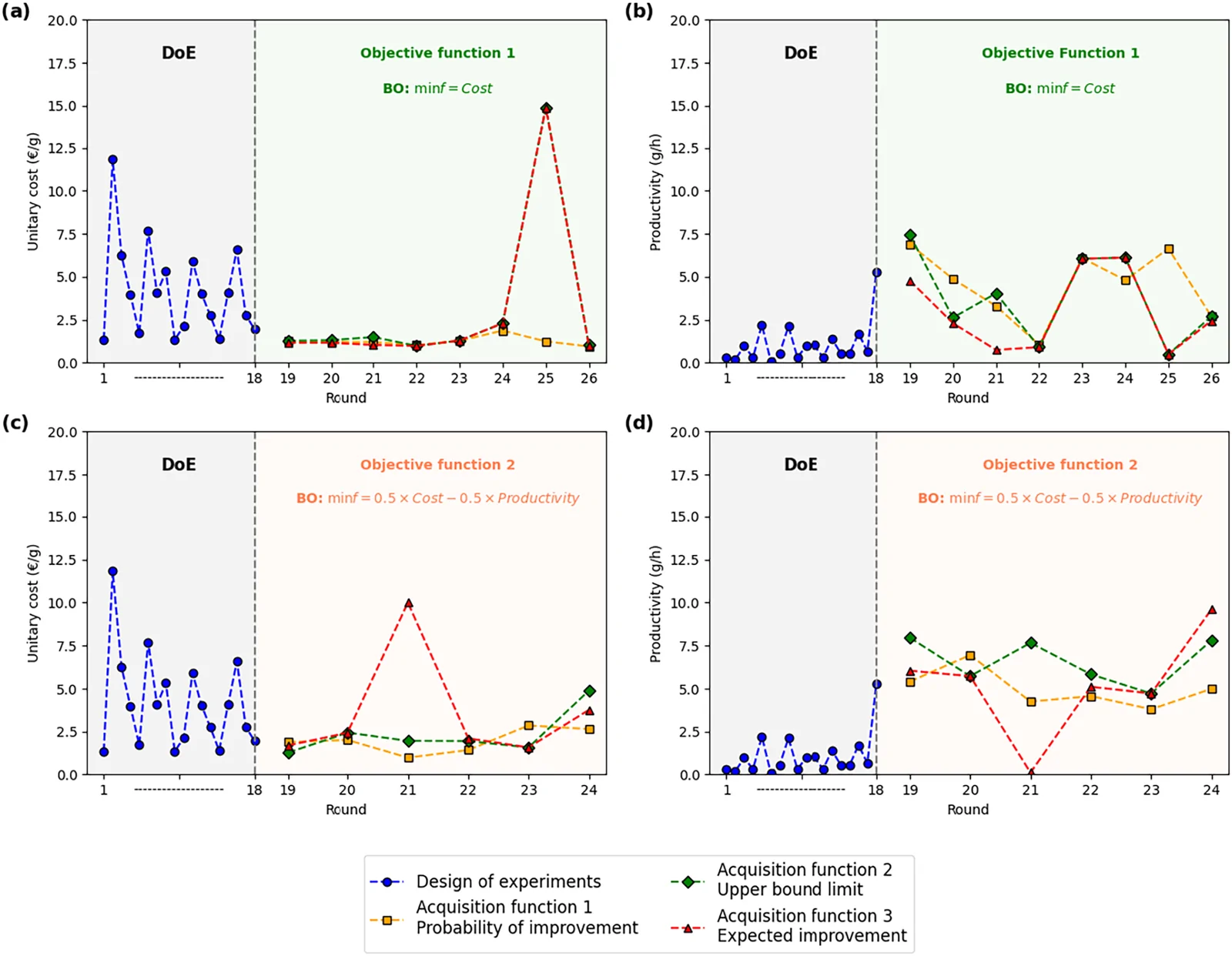
Evolution of Cost and Productivity during the Bayesian Optimization experiments. Graphs (a, b) display cost and productivity when using *OF*_1_ as target (minimize cost with penalization on low productivity). Graphs (c, d) show cost and productivity when using *OF*_2_ as target (minimize cost and maximize productivity, with the same weight). Blue points 1–18 correspond to the initial Design of Experiments, while the rest correspond to the points suggested by the different acquisition functions. More information about the data points can be found in Tables S2 and S3 in the Supporting Information.

With the LEI acquisition function, the algorithm found conditions where a high excess of ligand (PPh_3_) compared to the catalyst would lead to a steep decrease in yield (and therefore an increase in both cost and productivity) if temperature was not high enough, as shown by the offset points in Experiment 25 (*OF*_1_) and in Experiment 21 (*OF*_2_). This is usually related to the fact that under certain conditions, an excess of ligand can stabilize unreactive metal complexes or inhibit specific steps of the catalytic cycle that require ligand dissociation.^44^ Based on how the algorithm works, which is by balancing exploration and exploitation, it is within normal behavior that regions of poor results are sometimes found.

The parameters with the highest effect on the objective functions were determined using a Random Forest Feature Importance model included in scikit learn. The model identified residence time, the mol % of Pd­(OAc)_2_ and the solvent volumes as the parameters with the highest effect on the objective functions. The space explored for each of them is shown in the Supporting Information (Figure S1). In general, lower costs were achieved with lower solvent volumes and lower catalyst loadings. In the case of productivity, it benefitted from lower residence times, lower solvent volumes and higher catalyst loading. Finally, the PMI benefitted from lower amounts of solvent and moderate residence time and catalyst loading. This led the algorithm to exploit the volume between 1 and 3 min of residence time, 5 and 10 solvent volumes and 0.5–1 mol % of Pd­(OAc)_2_ to search for the optimal solution.

After the application of the BO algorithm, where productivity and unitary cost were considered the main objectives to optimize, we obtained a bivariable Pareto frontier, as shown in Figure 5 (see data in Table S4). All points in the Pareto frontier are equally optimal, since it is impossible to improve one of the objectives without worsening the other. However, it is important to note that in the case of 3 solvent volumes (marked in the Figure), sodium bromide precipitated in the reaction media. Although the reactor did not clog, this situation is not desirable when scaling-up, so this point should be disregarded as an optimal from an operational perspective.

**Figure 5:**
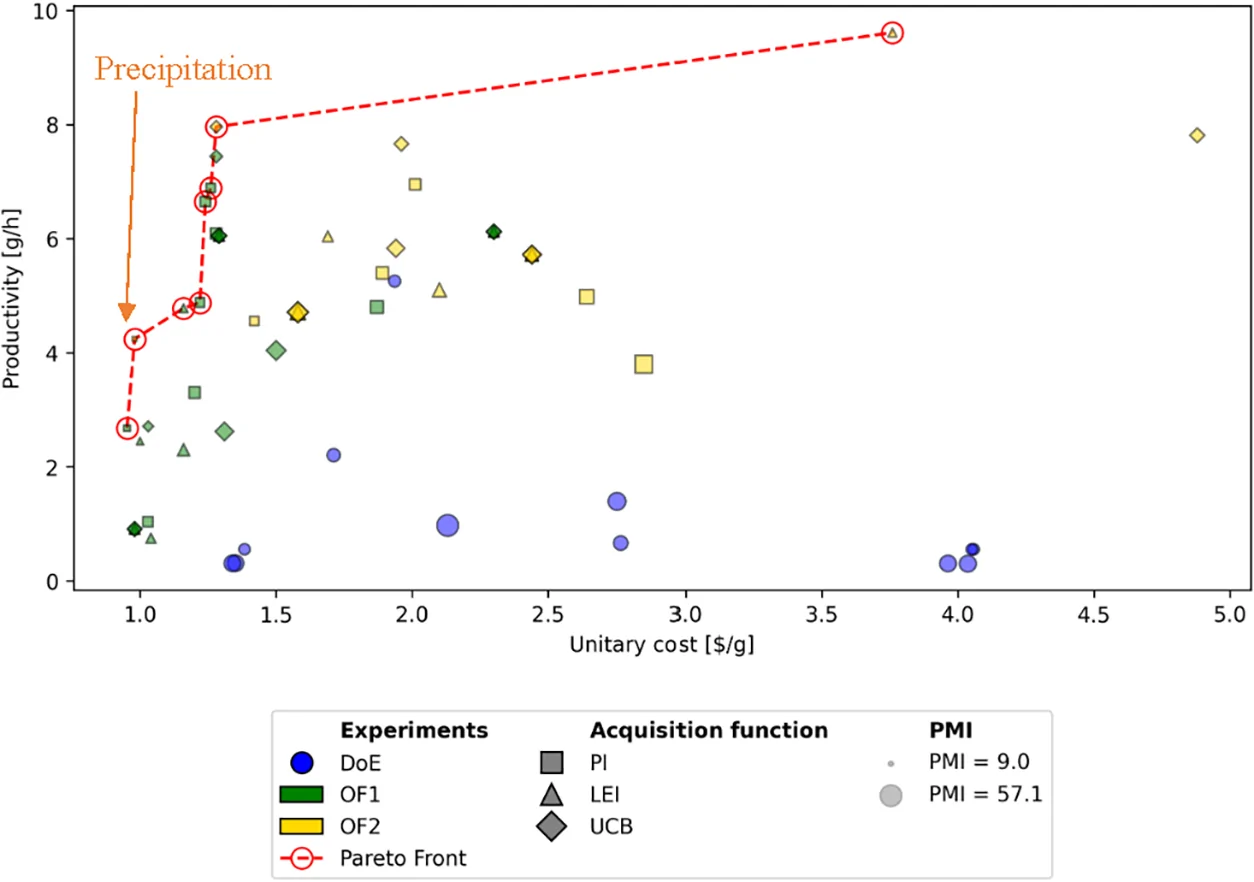
Pareto frontier of Productivity vs Cost for the Suzuki coupling reaction.

Notably, both objective functions proved useful in identifying optimal solutions within the Pareto plot. While both contributed to the generation of Pareto-optimal points, *OF*_1_ appeared more frequently among solutions associated with lower costs while *OF*_2_ was often associated with higher productivities. Similarly, both PI, LEI and UCB contributed to the obtention of optimal points, as shown in the Pareto Plot. Although LEI and UCB sometimes provided similar points to explore, the combination of the three acquisition functions has allowed for a faster exploration of the reaction space in terms of number of experiments and time needed to run them.

The PMI for each of the cases was calculated to assess the environmental efficiency of the process (as the process was not at industrial scale and there was no data for recycling or recuperation, no recuperation was considered for the time being). All of the PMI values in the optimal operation conditions fell within 9–18, a much lower rank than the one for the initial DoE, which went from 20 to around 60. This shows how, through the optimization of cost and productivity, PMI has also improved since it is directly related to the costs of the process. The current values would likely be lower after scale-up, when recycling and recuperation processes can be applied. Nonetheless, the values are already in the range of 10–40 per step, an interval considered appropriate by Boehringer Ingelheim.^41^

Of all the Pareto points shown, the authors considered the one with the minimal cost to be the most interesting one (cost of 0.95 $/g, productivity of 2.7 g/h), as it also corresponded to the conditions of lowest PMI. Despite productivity being low-to-moderate, it can be increased by increasing the flow rate and using bigger reactors accordingly. Therefore, the reaction was scaled up by a factor of 5 (keeping the characteristic dimensions of the reactor the same) to prove that the results achieved in the 1 mL reactor at the laboratory scale would be maintained for larger scales. The reaction yield was kept equal to the previous experiments, providing 5 times higher productivity (from 2.7 g/h to 14 g/h) while maintaining the lowest unitary cost (0.95 $/g) (Figure 6).

**Figure 6:**
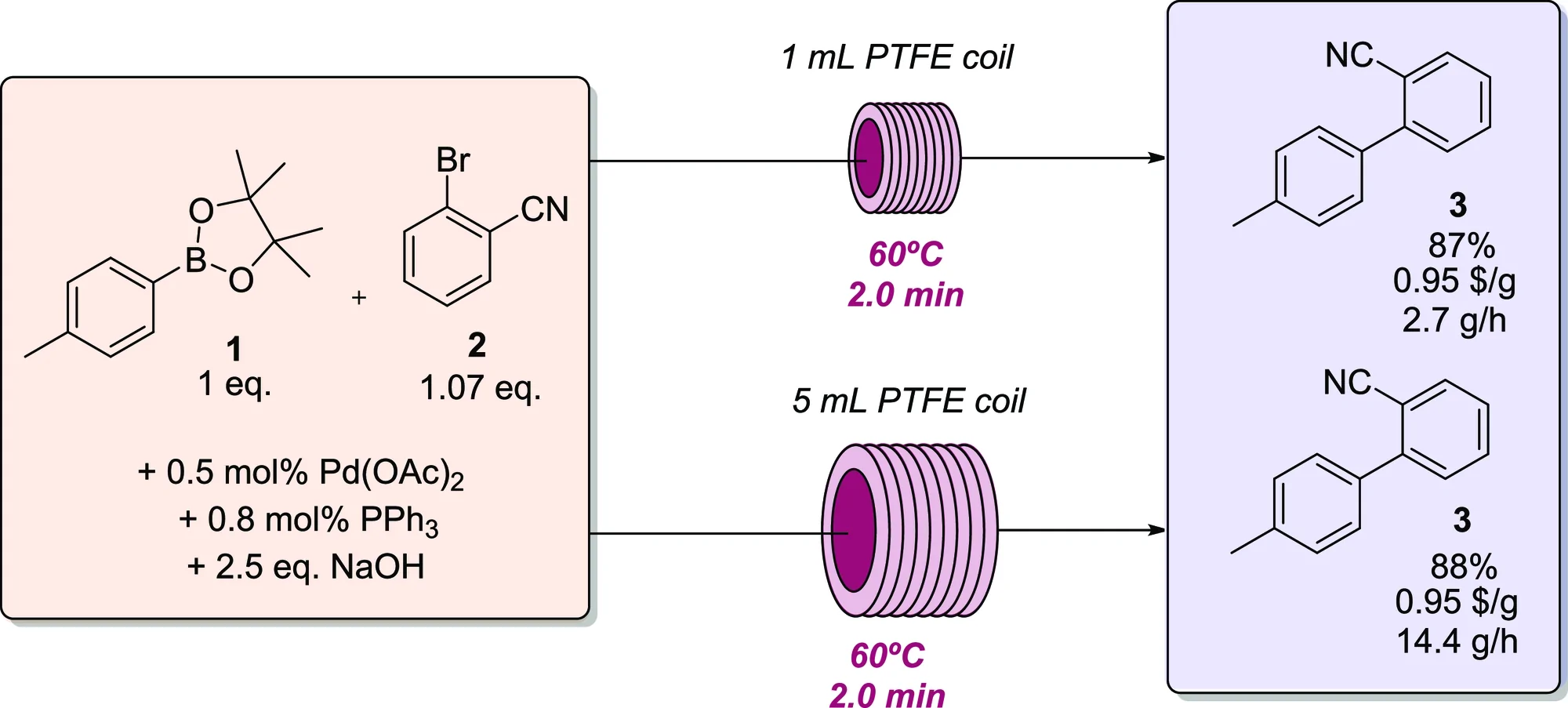
Scale-up proof-of-concept of the Suzuki reaction under optimal reaction conditions.

## 4 Conclusions

Bayesian Optimization algorithms have been applied to the very well-known Suzuki coupling to produce the biphenyl intermediate of sartans. Given the fact that it is a catalytic biphasic reaction with at least 6 variables involved, typical chemical methods to optimize reactions such as kinetic modeling or DoE techniques were not considered suitable. However, the implementation of an initial DoE followed by the BO algorithm led to an increase in productivity of up to 4 times and a reduction of cost around 30% with less overall experiments than a complete DoE and the pertinent response surfaces.

Several optimal points for operation were provided in a Pareto front, displaying the conditions which provide the lowest costs for each productivity. The point which provided the lowest cost and the lowest PMI was scaled up by a factor of 5 with the same yield, unitary cost and 5 times higher productivity, showing that the results obtained would also be applicable on other scales. Considering the recent results, this methodology could be applied to other complex reactions such as solid–liquid–gas systems, adding more restrictions in terms of environmental impact or cost.

## Supplementary Material



## Data Availability

The data that supports the findings of this study are available in the Supporting Information of this article.

## Notes

The values of individual costs, BO data points, and the ^1^H NMR spectrum of the 5x scale experiment (PDF)

## References

[1] ECHEMI . Valsartan Tops Global Consumption of Sartans, Arisartan Shows Highest Growth Rate 2023 https://www.echemi.com/cms/1561049.html. (accessed November 3, 2025).

[2] Dézsi C. A. (2016). The different
therapeutic choices with ARBs. Which one to give? When? Why?. AAm. J. Cardiovasc. Drugs.

[3] Georgiou N., Chontzopoulou E., Routsi E. A., Stavrakaki I. G., Petsas E., Zoupanou N., Kakava M. G., Tzeli D., Mavromoustakos T., Kiriakidi S. (2024). Exploring Hypertension: The Role
of AT1 Receptors, Sartans, and Lipid Bilayers. ACS Omega.

[4] Pati H. N., Vangala V. B., Hindupur R. M. (2014). A Review on Synthesis of Antihypertensive
Sartan Drugs. Int. J. Pharma Res. Rev..

[5] Veera Reddy, A. ; Bhaskara Rao, S. U. ; Rajendiran, C. ; Jasti, V. Process for the preparation of Losartan. US Patent US7,914,425B2, 2011.

[6] Nisnevich, G. ; Rukhman, I. ; Pertsikov, N. ; Kaftanov, J. ; Dolitzky, B.-Z. Synthesis of Irbesartan. US Patent US7,019,148B2, 2006.

[7] Dézsi C. A. (2016). The Different
Therapeutic Choices with ARBs. Which One to Give? When? Why?. Am. J. Cardiovasc. Drugs.

[8] Veverka, M. ; Putala, M. ; Brath, H. ; Zuppancic Process for the preparation of Sartan Derivatives and Intermediates Useful in Such Process. US Patent US7,868,180B2, 2011.

[9] Larsen R. D., King A. O., Chen C. Y., Corley E. G., Foster B. S., Roberts F. E., Yang C., Lieberman D. R., Reamer R. A., Tschaen D. M., Verhoeven T. R., Reider P. J., Lo Y. S., Rossano L. T., Brookes A. S., Meloni D., Moore J. R., Arnett J. F. (1994). Efficient Synthesis
of Losartan, A Nonpeptide Angiotensin II Receptor Antagonist. J. Org. Chem..

[10] Jana A., Ravichandiran V., Swain S. P. (2021). Application of organometallic catalysts
for the synthesis of o-tolyl benzonitrile, a key starting material
for sartans. New J. Chem..

[11] Goossen L. J., Melzer B. (2007). Synthesis of Valsartan
via Decarboxylative Biaryl Coupling. J. Org.
Chem..

[12] Kumar A. S., Ghosh S., Mehta G. N. (2010). Efficient
and improved synthesis
of Telmisartan. Beilstein J. Org. Chem..

[13] Kadu B. S. (2021). Suzuki–Miyaura
cross coupling reaction: recent advancements in catalysis and organic
synthesis. Catal. Sci. Technol..

[14] Miyaura N., Yamada K., Suzuki A. (1979). A new stereospecific
cross-coupling
by the palladium-catalyzed reaction of 1-alkenylboranes with 1-alkenyl
or 1-alkynyl halides. Tetrahedron Lett..

[15] Fantoni T., Palladino C., Grigolato R., Muzzi B., Ferrazzano L., Tolomelli A., Cabri W. (2025). Mastering palladium-catalyzed cross-coupling
reactions: the critical role of in situ pre-catalyst reduction design. Org. Chem. Front..

[16] Chung J. Y. L., Cvetovich R. J., McLaughlin M., Amato J., Tsay F. R., Jensen M., Weissman S., Zewge D. (2006). Synthesis of a naphthyridone
p38 MAP kinase inhibitor. J. Org. Chem..

[17] Kerdesky F. A. J., Leanna M. R., Zhang J., Li W., Lallaman J. E., Ji J., Morton H. E. (2006). An efficient multikilogram
synthesis of ABT-963: A
selective COX-2 inhibitor. Org. Process Res.
Dev..

[18] Williams M.
J., Chen Q., Codan L., Dermenjian R. K., Dreher S., Gibson A. W., He X., Jin Y., Keen S. P., Lee A. Y., Lieberman D. R., Lin W., Liu G., McLaughlin M., Reibarkh M., Scott J. P., Strickfuss S., Tan L., Varsolona R. J., Wen F. (2016). Process Development of the HCV NS5B Site D Inhibitor MK-8876. Org. Process Res. Dev..

[19] Kuethe J. T., Journet M., Peng Z., Zhao D., McKeown A., Humphrey G. R. (2016). Development of a Multikilogram Scale
Synthesis of a
TRPV1 Antagonist. Org. Process Res. Dev..

[20] Zhao M. M., Zhang H., Iimura S., Bednarz M. S., Kanamarlapudi R. C., Yan J., Lim N. K., Wu W. (2020). Process Development of Tryptophan
Hydroxylase Inhibitor LX1031, a Drug Candidate for the Treatment of
Irritable Bowel Syndrome. Org. Process Res.
Dev..

[21] Yamashita Y., Morinaga Y., Kasai M., Hashimoto T., Takahama Y., Ohigashi A., Yonishi S., Akazome M. (2017). A Practical
and Scalable Synthesis of a Glucokinase Activator via Diastereomeric
Resolution and Palladium-Catalyzed C-N Coupling Reaction. Org. Process Res. Dev..

[22] Taylor C. J., Pomberger A., Felton K. C., Grainger R., Barecka M., Chamberlain T. W., Bourne R. A., Johnson C. N., Lapkin A. A. (2023). A Brief
Introduction to Chemical Reaction Optimization. Chem. Rev..

[23] Jones D. R., Schonlau M., Welch W. J. (1998). Efficient Global Optimization of
Expensive Black-Box Functions. J. Global Optim..

[24] Wu Y., Walsh A., Ganose A. M. (2024). Race to the bottom: Bayesian optimization
for chemical problems. Digital Discovery.

[25] Taylor C. J., Felton K. C., Wigh D., Jeraal M. I., Grainger R., Chessari G., Johnson C. N., Lapkin A. A. (2023). Accelerated Chemical
Reaction Optimization Using Multi-Task Learning. ACS Cent. Sci..

[26] Braconi E., Godineau E. (2023). Bayesian Optimization as a Sustainable
Strategy for
Early-Stage Process Development? A Case Study of Cu-Catalyzed C-N
Coupling of Sterically Hindered Pyrazines. ACS
Sustainable Chem. Eng..

[27] Wagner F. L., Sagmeister P., Tampone T. G., Manee V., Yerkozhanov D., Buono F. G., Williams J. D., Kappe C. O. (2024). Self-Optimizing
Flow Reactions for Sustainability: An Experimental Bayesian Optimization
Study. ACS Sustainable Chem. Eng..

[28] Liang R., Hu H., Han Y., Chen B., Yuan Z. (2024). CAPBO: A cost-aware
parallelized Bayesian optimization method for chemical reaction optimization. AIChE J..

[29] Liang R., Duan X., Zhang J., Yuan Z. (2022). Bayesian based reaction
optimization for complex continuous gas-liquid-solid reactions. React. Chem. Eng..

[30] Kwon Y., Lee D., Kim J. W., Choi Y. S., Kim S. (2022). Exploring Optimal Reaction
Conditions Guided by Graph Neural Networks and Bayesian Optimization. ACS Omega.

[31] Park S., Na J., Kim M., Lee J. M. (2018). Multi-objective
Bayesian optimization
of chemical reactor design using computational fluid dynamics. Comput. Chem. Eng..

[32] Fan S., Zhang X., Hong X., Liao Z., Chen Y., Ren C., Yang Y., Wang J., Yang Y. (2024). Kinetic Parameter Estimation
of the Polyethylene Process by Bayesian Optimization. Ind. Eng. Chem. Res..

[33] Wang Y., Chen T. Y., Vlachos D. G. (2021). NEXTorch: A Design
and Bayesian Optimization
Toolkit for Chemical Sciences and Engineering. J. Chem. Inf. Model..

[34] Balandat, M. ; Karrer, B. ; Jiang, D. R. ; Daulton, S. ; Letham, B. ; Gordon, A. ; Bakshy, E. In BOTORCH: A Framework for Efficient Monte-Carlo Bayesian Optimization; Advances in Neural Information Processing Systems 33, Larochelle, H. ; Ranzato, M. ; Hadsell, R. ; Balcan, M. F. ; Lin, H. , Eds.; NIPS: Vancouver, 2020 https://proceedings.neurips.cc/paper_files/paper/2020/file/f5b1b89d98b7286673128a5fb112cb9a-Paper.pdf. pp 21524–21538, (accessed November 5, 2025)..

[35] Github . Why Ax?. https://ax.dev/docs/why-ax. (accessed November 5, 2025).

[36] Dürholt, J. P. ; Asche, T. S. ; Kleinekorte, J. ; Mancino-Ball, G. ; Schiller, B. S. S. ; Keupp, J. ; Osburg, A. ; Boyne, T. ; Misener, R. ; Eldred, R. ; Costa, W. S. ; Kappatou, C. ; Lee, R. M. ; Linzner, D. ; Walz, D. ; Wulkow, N. ; Shafei, B. BoFire: Bayesian Optimization Framework Intended for Real Experiments 2024 http://arxiv.org/abs/2408.05040.

[37] Biswas A., Liu Y., Creange N., Liu Y.-C., Jesse S., Yang J.-C., Kalinin S. V., Ziatdinov M. A., Vasudevan R. K. (2024). A dynamic
Bayesian optimized active recommender system for curiosity-driven
partially Human-in-the-loop automated experiments. NPJ. Comput. Mater..

[38] Sousa J., Sousa A., Brueckner F., Reis L. P., Reis A. (2025). Human-in-the-loop
Multi-objective Bayesian Optimization for Directed Energy Deposition
with in-situ monitoring. Robot. Comput. Integr.
Manuf..

[39] Hughes J. C., York D. J., Yager K. G., Osuji C. O., Composto R. J. (2026). Using Flory–Huggins-informed
human-in-the-loop Bayesian optimization to map the phase diagram of
polymer blends. Digital Discovery.

[40] Jimenez-Gonzalez C., Ponder C. S., Broxterman Q. B., Manley J. B. (2011). Using the right
green yardstick: Why process mass intensity is used in the pharmaceutical
industry to drive more sustainable processes. Org. Process Res. Dev..

[41] Dach R., Song J. J., Roschangar F., Samstag W., Senanayake C. H. (2012). The eight
criteria defining a good chemical manufacturing process. Org. Process Res. Dev..

[42] Hvarfner, C. ; Hellsten, E. O. ; Nardi, L. Vanilla Bayesian Optimization Performs Great in High Dimensions 2024 http://arxiv.org/abs/2402.02229.

[43] Ament, S. ; Daulton, S. ; Eriksson, D. ; Balandat, M. ; Bakshy, E. Unexpected Improvements to Expected Improvement for Bayesian Optimization 2025 http://arxiv.org/abs/2310.20708.

[44] Firsan S. J., Sivakumar V., Colacot T. J. (2022). Emerging Trends in Cross-Coupling:
Twelve-Electron-Based L1Pd(0) Catalysts, Their Mechanism of Action,
and Selected Applications. Chem. Rev..

