# Supplementary material for: Bayesian Optimization of a Suzuki Coupling for the Industrial Synthesis of a Sartan Drug Intermediate
Source: ACS Omega. 2026 Aug 3;11(32):48090–8. doi: 10.1021/acsomega.6c04219 (PMC13491879; doi:10.1021/acsomega.6c04219)

Supplementary Information

Bayesian optimization of a Suzuki Coupling for the Industrial Synthesis of a Sartan Drug Intermediate

Maite Molins,[a] Javier Fernandez-Garcia,[b] Daniel Vázquez,[b*] Xavier Berzosa [a*]

[a] *CRISOL Group, Dept. of Chemical Engineering and Materials Science. IQS-School of Engineering, Universitat Ramon Llull. Via Augusta 390, 08017 Barcelona, Spain.*

[b] *GESPA Group, Dept. of Chemical Engineering and Materials Science. IQS-School of Engineering, Universitat Ramon Llull. Via Augusta 390, 08017 Barcelona, Spain.*

**Corresponding authors: Xavier Berzosa (xavier.berzosa@iqs.url.edu), Daniel Vázquez (daniel.vazquez@iqs.url.edu)*

Funding: FPU-Spanish-Government PhD Scholarship contract (FPU21/05233)

Keywords: Bayesian optimization, C-C coupling, flow chemistry, machine learning, sartans, Suzuki-Miyaura cross-coupling.

1. Parameters for calculating cost and productivity

Table S1. Name, unitary cost and relevant properties of reagents and solvents. Costs checked on September 29th 2025.

Type	Name and SMILES	Cost	Reference
**Limiting reagent**	4,4,5,5-Tetramethyl-2-(*p*-tolyl)-1,3,2-dioxaborolane B1(OC(C(O1)(C)C)(C)C)C2=CC=C(C=C2)C	353.64 $/g	https://www.1clickchemistry.com/product/MUMxNzc3NQ==
**Reagent**	*o*-bromobenzonitrile C1=CC=C(C(=C1)C#N)Br	137 $/g	https://www.chemicalbook.com/SupplierPriceList_EN.aspx?cbn=CB6439565#price
**Reagent**	$Pd(OAc)_2$	46.83 $/g	https://www.chemicalbook.com/SupplierPriceList_EN.aspx?cbn=CB1187458&c=100g#price
**Reagent**	PPh_3 C1=CC=C(C=C1)P(C2=CC=CC=C2)C3=CC=CC=C3. C1=CC=C(C=C1)P(C2=CC=CC=C2)C3=CC=CC=C3	31 $/kg	https://www.chemicalbook.com/SupplierPriceList_EN.aspx?cbn=CB8771461&c=25KG#price
**Solvent**	THF	2041 $/MT	https://www.imarcgroup.com/tetrahydrofuran-pricing-report
**Reagent**	NaOH	1.22 $/g	https://www.chemicalbook.com/SupplierPriceList_EN.aspx?cbn=CB8105015&c=100KG#price
**Solvent**	Water	0.6 $/MT	Anand K. Plappally & John H. Lienhard V (2012): Costs for water supply, treatment, end-use and reclamation, Desalination and Water Treatment, DOI:10.1080/19443994.2012.708996

2. BO progression

The objective functions OF_1 and OF_2 were defined:

$$OF_1(x) = \begin{cases} Cost & P \geq 3\ g/h \\ 10\ \$/g & P < 3 g/h \end{cases} \qquad\qquad OF_2(x) = Cost - P$$

OF_1 represents the unitary cost $\left[\frac{\$}{g}\right]$ with a penalization on productivities $P\left[\frac{g}{h}\right]$ lower than 3 g/h by introducing a large value in the objective function (10 $/g). OF_2 consisted in a weighted average approach including both unitary cost and productivity with the same weights. The equation was written in such a way that an increase in productivity and a decrease in cost would lead to the minimization of OF_2.

In the case of OF_1, before applying the penalty it was necessary to have enough points that satisfied the condition. If not, all values would be 10$/g and no tendencies would be perceived by the optimization algorithm. This is why the penalty was not included until 7 points that satisfied the conditions had been found (Round 23). Until then, only the minimization of cost was considered.

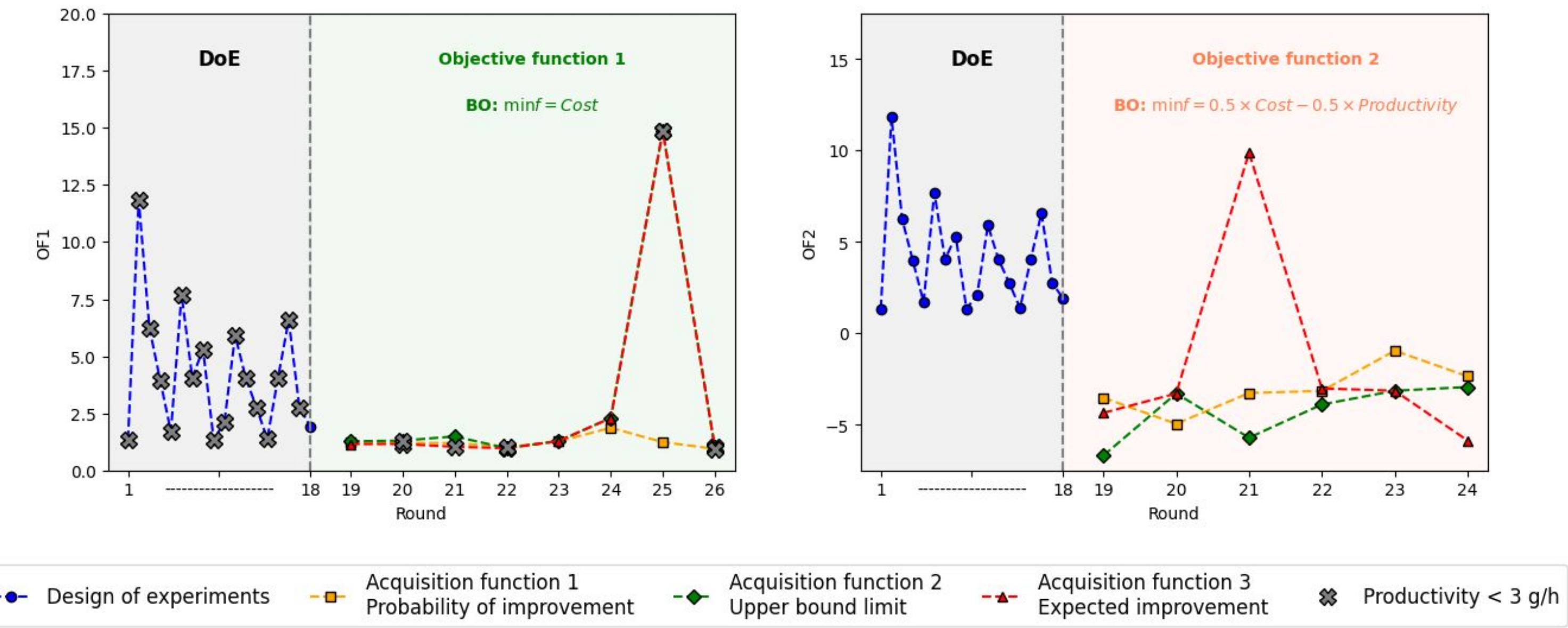

Figure S1. Progression of the Objective Functions OF1 and OF2 along the BO rounds.

3. BO data tracking

3.1. OF_1

$$OF_1(x) = \begin{cases} Cost & P \geq 3\ g/h\ or\ \#Round < 23 \\ 10 & P < 3g/h\ and\ \#Round \geq 23 \end{cases}$$

Table S2. All points involved in the Bayesian Optimization using the objective function OF1.

Exp	Design / Acq. Func.	Res. time (min)	T (°C)	Equiv. 2-BrBN	Equiv. $Pd(OAc)_2$	Equiv. PPh_3	V dte.	Yield	Cost ($/g)	Prod. (g/h)	OF_1 pred.	OF_1	PMI	Obs.
1	DoE	5	50	1.2	0.01	0.01	20	85%	1.35	0.31	-	1.35 / 10	35.7	
2	DoE	1	50	1.2	0.01	0.05	20	10%	11.84	0.18	-	11.8 / 10	313	
3	DoE	1	50	1.05	0.05	0.01	20	53%	6.24	0.97	-	6.24 / 10	57.1	
4	DoE	5	50	1.05	0.05	0.05	20	83%	3.96	0.31	-	3.96 / 10	36.3	
5	DoE	1	50	1.05	0.01	0.01	10	65%	1.71	2.20	-	1.71 / 10	25.3	
6	DoE	5	50	1.05	0.01	0.05	10	14%	7.70	0.10	-	7.70 / 10	114	
7	DoE	5	50	1.2	0.05	0.01	10	81%	4.06	0.56	-	4.05 / 10	20.3	
8	DoE	1	50	1.2	0.05	0.05	10	62%	5.32	2.12	-	5.32 / 10	26.6	
9	DoE	5	70	1.05	0.01	0.01	20	84%	1.34	0.31	-	1.34 / 10	35.9	
10	DoE	1	70	1.05	0.01	0.05	20	53%	2.13	0.97	-	2.13 / 10	57.1	
11	DoE	1	70	1.2	0.05	0.01	20	56%	5.91	1.03	-	5.91 / 10	54.0	
12	DoE	5	70	1.2	0.05	0.05	20	82%	4.04	0.30	-	4.04 / 10	36.9	
13	DoE	1	70	1.2	0.01	0.01	10	41%	2.75	1.39	-	2.75 / 10	40.3	
14	DoE	5	70	1.2	0.01	0.05	10	81%	1.38	0.56	-	1.38 / 10	20.3	
15	DoE	5	70	1.05	0.05	0.01	10	81%	4.05	0.55	-	4.05 / 10	20.2	
16	DoE	1	70	1.05	0.05	0.05	10	50%	6.58	1.70	-	6.58 / 10	32.9	
17	DoE	3	60	1.125	0.03	0.03	15	80%	2.77	0.66	-	2.77 / 10	29.1	
18	DoE	0.5	50	1.05	0.005	0.010	5	42%	1.94	5.25	-	1.94	22.1	
19	PI	0.54	54	1.07	0.005	0.010	5.5	65%	1.26	6.89	2.24 ± 1.24	1.26	15.5	Start BO
19	UCB	0.5	52	1.06	0.005	0.010	5.4	64%	1.28	7.44	2.16 ± 0.68	1.28	15.5	
19	LEI	0.8	57	1.09	0.005	0.010	5.8	71%	1.16	4.78	2.44 ± 1.97	1.16	14.8	

20	PI	0.7	55	1.08	0.0054	0.010	6.2	70%	1.22	4.88	0.91 ± 0.25	1.22	15.8	Min equiv. PPh_3 to 0.008
20	UCB	0.84	56	1.08	0.0052	0.008	10	65%	1.31	2.62	0.76 ± 1.07	1.31 / 10	25.3	
20	LEI	1.06	59	1.05	0.005	0.008	10	71%	1.16	2.30	1.17 ± 1.64	1.16 / 10	22.8	
21	PI	0.8	56	1.08	0.0054	0.009	9	71%	1.20	3.30	0.89 ± 0.22	1.20	21.0	
21	UCB	0.5	54	1.08	0.005	0.008	9.4	56%	1.50	4.04	1.06 ± 0.59	1.50	27.8	
21	LEI	4.3	67	1.17	0.0072	0.024	10	93%	1.04	0.75	2.48 ± 2.41	1.04 / 10	17.7	
22	PI	3	59	1.12	0.007	0.009	10	92%	1.03	1.04	0.74 ± 1.74	1.03 / 10	17.7	
22	UCB/LEI	3.4	59	1.12	0.006	0.008	10	91%	0.98	0.91	0.82 ± 1.92	0.98 / 10	17.9	
23	PI	0.5	53	1.07	0.005	0.010	7.2	65%	1.28	6.09	0.07 ± 0.28	1.28	19.2	
23	UCB/LEI	0.5	52	1.12	0.005	0.008	7	65%	1.29	6.05	-2.20 ± 1.51	1.29	18.9	
24	PI	0.5	55	1.08	0.005	0.009	5.9	44%	1.87	4.80	-0.31 ± 0.27	1.87	24.2	
24	UCB/LEI	0.5	59	1.09	0.015	0.008	6.3	60%	2.30	6.12	-1.70 ± 1.83	2.30	18.8	
25	PI	0.5	54	1.08	0.005	0.009	6.8	67%	1.24	6.65	0.48 ± 0.16	1.24	17.9	
25	UCB/LEI	0.5	56	1.1	0.005	0.021	7.4	6%	14.87	0.49	-0.41 ± 2.09	14.9 / 10	228	
26	PI	2	60	1.07	0.005	0.008	5	87%	0.952	2.68	-1.13 ± 1.61	0.95 / 10	10.9	
26	UCB	1.67	56	1.16	0.005	0.008	5.7	81%	1.03	2.71	2.93 ± 2.09	1.03 / 10	12.9	
26	LEI	1.91	56	1.16	0.005	0.008	5.6	84%	1.00	2.45	2.79 ± 2.21	1.00 / 10	12.3	

3.2. OF_2

$$OF_2(x) = Cost - P$$

Table S3. All points involved in the Bayesian Optimization using the objective function OF2.

Exp	Design / Acq. Func.	Res. time (min)	T (°C)	Equiv. 2-BrBN	Equiv. $Pd(OAc)_2$	Equiv. PPh_3	V dte.	Yield	Cost ($/g)	Prod. (g/h)	OF_2 pred.	OF_2	PMI	Obs.
1	DoE	5	50	1.2	0.01	0.01	20	85%	1.35	0.31	-	1.04	35.7	
2	DoE	1	50	1.2	0.01	0.05	20	10%	11.84	0.18	-	9.82	313	
3	DoE	1	50	1.05	0.05	0.01	20	53%	6.24	0.97	-	5.27	57.1	
4	DoE	5	50	1.05	0.05	0.05	20	83%	3.96	0.31	-	3.66	36.3	
5	DoE	1	50	1.05	0.01	0.01	10	65%	1.71	2.20	-	-0.49	25.3	
6	DoE	5	50	1.05	0.01	0.05	10	14%	7.70	0.10	-	7.60	114	
7	DoE	5	50	1.2	0.05	0.01	10	81%	4.06	0.56	-	3.50	20.3	
8	DoE	1	50	1.2	0.05	0.05	10	62%	5.32	2.12	-	3.20	26.6	
9	DoE	5	70	1.05	0.01	0.01	20	84%	1.34	0.31	-	1.03	35.9	
10	DoE	1	70	1.05	0.01	0.05	20	53%	2.13	0.97	-	1.16	57.1	
11	DoE	1	70	1.2	0.05	0.01	20	56%	5.91	1.03	-	4.88	54.0	
12	DoE	5	70	1.2	0.05	0.05	20	82%	4.04	0.30	-	3.73	36.9	
13	DoE	1	70	1.2	0.01	0.01	10	41%	2.75	1.39	-	1.36	40.3	
14	DoE	5	70	1.2	0.01	0.05	10	81%	1.38	0.56	-	0.83	20.3	
15	DoE	5	70	1.05	0.05	0.01	10	81%	4.05	0.55	-	3.50	20.2	
16	DoE	1	70	1.05	0.05	0.05	10	50%	6.58	1.70	-	4.88	32.9	
17	DoE	3	60	1.125	0.03	0.03	15	80%	2.77	0.66	-	2.10	29.1	
18	DoE	0.5	50	1.05	0.005	0.010	5	42%	1.94	5.25	-	-3.32	22.1	
19	PI	0.5	53	1.07	0.005	0.010	5.5	44%	1.89	5.40	-2.61 ± 1.15	-3.52	21.6	Start BO
19	UCB	0.5	51	1.05	0.005	0.010	5.4	64%	1.28	7.96	-2.98 ± 0.38	-6.68	14.6	
19	LEI	0.5	55	1.08	0.005	0.010	5.8	49%	1.69	6.04	-2.04 ± 1.70	-4.34	19.3	
20	PI	0.4	55	1.05	0.004	0.010	6.2	38%	2.01	6.95	-10.39 ± 1.11	-4.94	20.6	Min residence time to 0.4 Min equiv. $Pd(OAc)_2$ to 0.004

														Min equiv. PPh_3 to 0.008
20	UCB/LEI	0.4	57	1.05	0.004	0.010	10	31%	2.44	5.72	-8.43 ± 2.14	-3.28	25.0	
21	PI	1.44	53	1.05	0.003	0.008	10	72%	0.98	4.24	-3.40 ± 1.60	-3.26	9.0	Min equiv. $Pd(OAc)_2$ to 0.003
21	UCB	0.4	52	1.05	0.003	0.008	9	36%	1.96	7.66	-4.12 ± 1.41	-5.70	17.9	
21	LEI	0.4	52	1.05	0.003	0.025	9.4	0%	10.00	0.11	-2.60 ± 2.91	9.89	1302	
22	PI	0.8	54	1.05	0.0087	0.008	10	72%	1.42	4.56	-5.55 ± 1.24	-3.14	15.7	
22	UCB	0.4	56	1.05	0.0103	0.008	10	57%	1.94	5.83	-6.65 ± 1.76	-3.88	24.0	
22	LEI	0.4	56	1.05	0.0089	0.008	10	50%	2.10	5.10	-6.79 ± 1.73	-3.00	27.6	
23	PI	0.4	50	1.05	0.0034	0.006	7.2	26%	2.85	3.80	-6.82 ± 0.92	-0.94	39.1	Min equiv. PPh_3 to 0.006
23	UCB/LEI	0.4	50	1.05	0.003	0.006	7	46%	1.58	4.71	-6.9 ± 0.87	-3.13	29.9	
24	PI	0.4	54	1.05	0.0052	0.009	5.9	31%	2.64	4.98	-5.42 ± 0.25	-2.34	28.5	
24	UCB	0.4	52	1.05	0.0223	0.008	6.3	36%	4.88	7.81	-3.27 ± 1.86	-2.93	18.5	
24	LEI	0.4	52	1.05	0.0206	0.007	6.8	44%	3.76	9.61	-2.90 ± 1.65	-5.85	15.0	

4. Effect of variables on the objective functions

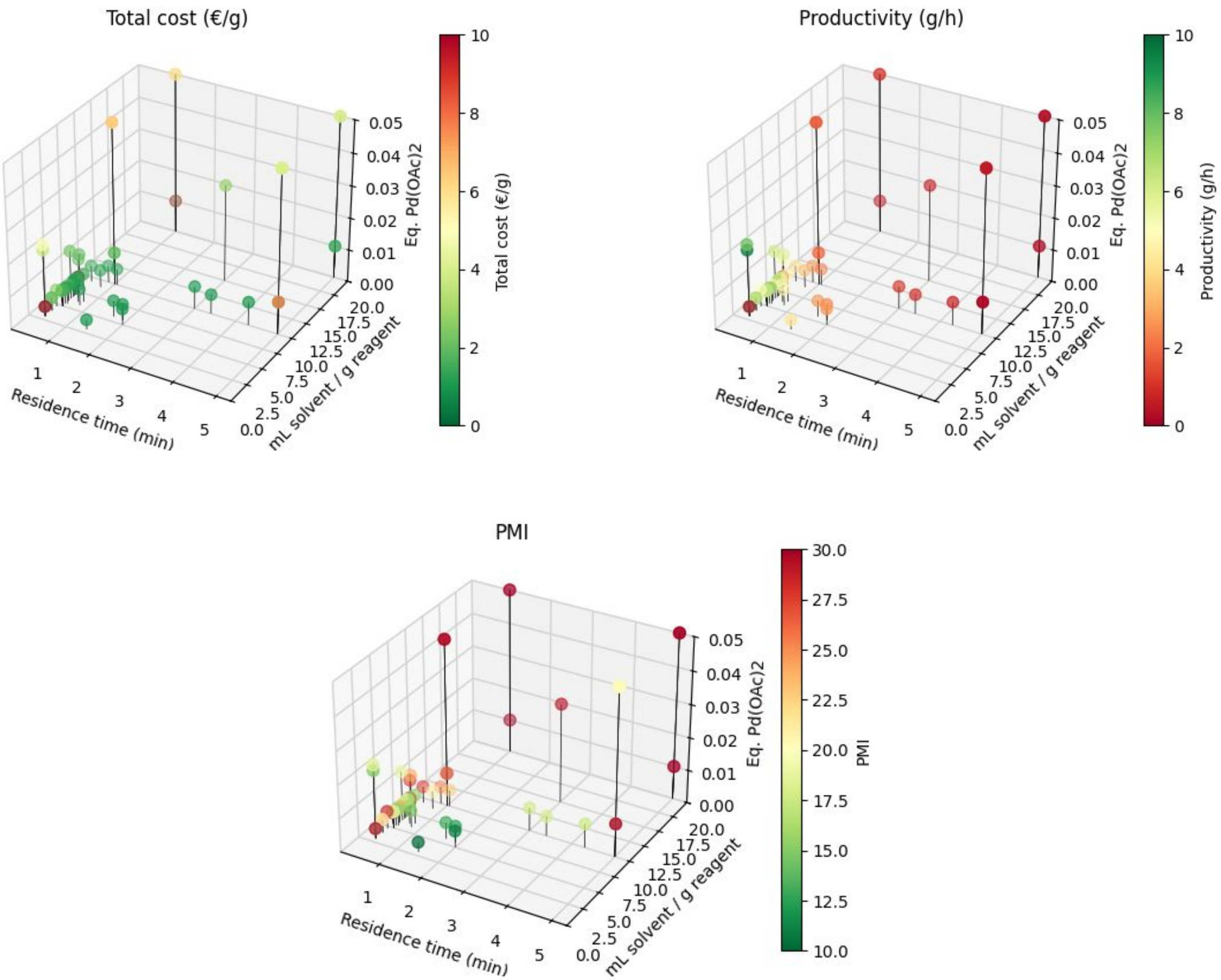

Figure S2. The variables with the highest effect on the objective functions: residence time, equivalents of $Pd(OAc)_2$ and mL of solvent per gram of limiting reagent and their effect on the process metrics.

5. Pareto Front

Table S4. Data displayed in the Pareto front. Pareto points are displayed in red.

Unitary cost ($/g)	Productivity (g/h)	PMI
1.26	6.89	15.5
1.28	7.44	15.5
1.16	4.78	14.8
1.21	4.88	15.8
1.31	2.62	25.3
1.16	2.30	22.8
1.20	3.30	21.0
1.50	4.04	27.8
1.04	0.75	17.7
1.03	1.04	17.7
0.98	0.91	17.9
1.27	6.09	19.2
1.29	6.05	18.9
1.87	4.80	24.2
2.30	6.12	18.8
1.24	6.65	17.9
0.95	2.68	10.9
1.03	2.71	12.9
1.00	2.45	12.3
1.24	6.65	17.4
1.89	5.40	21.6
1.28	7.96	14.6
1.69	6.04	19.3

2.01	6.95	20.6
**Unitary cost ($/g)**	**Productivity (g/h)**	**PMI**
2.44	5.72	25.0
0.98	4.24	8.97
1.96	7.66	17.9
1.42	4.56	15.7
1.94	5.83	24.0
2.10	5.10	27.6
2.85	3.80	39.1
1.58	4.71	29.9
2.64	4.98	28.5
4.88	7.81	18.5
3.76	9.61	15.0

6. ¹H-NMR spectrum of 5x scale experiment

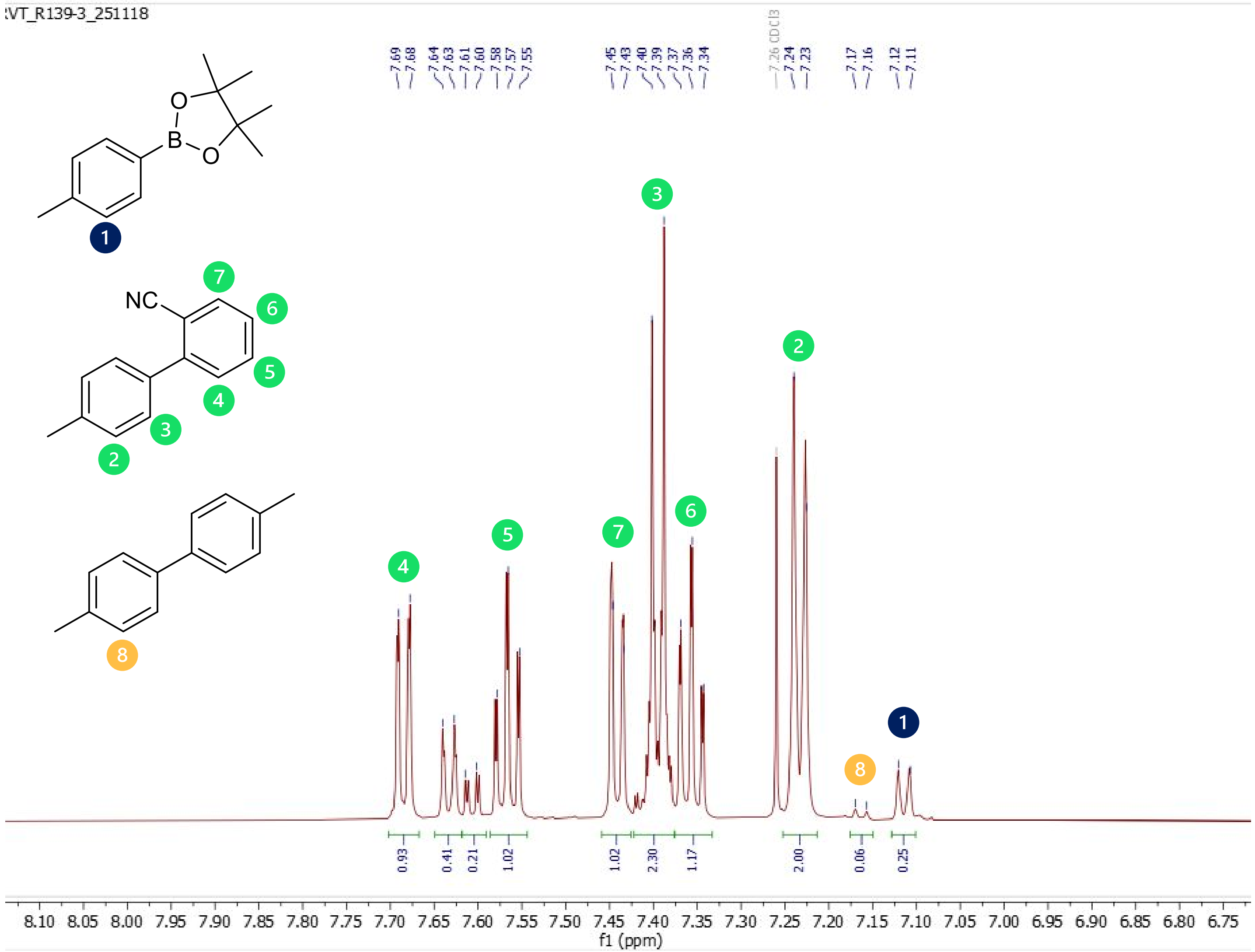

Supplement: Supplementary file 1 [file ao6c04219_si_001.pdf]
